# IL-18 Serum Level in Adult Onset Still's Disease: A Marker of Disease Activity

**DOI:** 10.1155/2012/156890

**Published:** 2012-06-18

**Authors:** Serena Colafrancesco, Roberta Priori, Cristiano Alessandri, Carlo Perricone, Monica Pendolino, Giovanna Picarelli, Guido Valesini

**Affiliations:** Reumatologia, Dipartimento di Medicina Interna e Specialità Mediche, Sapienza Università di Roma, Viale del Policlinico 155, 00161 Roma, Italy

## Abstract

*Introduction*. Immunological factors seem to play a pivotal role in Adult Onset Still's Disease (AOSD). Among all, IL-18 cytokine is overexpressed and drives the inflammatory process. *Objective*. We aimed to investigate the levels of IL-18 in sera of Italian patients with AOSD and to assess its possible role as a marker of disease activity. *Methods*. IL-18 serum levels were determined by ELISA in 26 Italian patients with AOSD. Disease activity was assessed using Pouchot's criteria. As controls, 21 patients with Rheumatoid Arthritis (RA), 21 patients with Sjogren's Syndrome (SS), 20 patients with Systemic Lupus Erythematosus (SLE), and 21 healthy subjects (normal human sera, NHS) were evaluated. *Results*. IL-18 serum levels were significantly higher in patients with active AOSD than in non-active (*P* = 0.001) and control groups (RA *P* = 0.0070, SS *P* = 0.0029, SLE *P* = 0.0032, NHS *P* = 0.0004). A significant correlation between IL-18 serum levels and disease activity (*P* < 0.0001), and laboratory parameters as ferritin (*P* = 0.0127) and C-reactive protein (*P* = 0.0032) was demonstrated. *Conclusions*. Higher levels of IL-18 are detected in active AODS patients and correlate with disease activity and inflammatory laboratory features. ROC-AUC analysis of the serum concentration of IL-18 suggests that it can be considered a diagnostic marker of AOSD. This paper supports the targeting of this cytokine as a possible therapeutic option in AOSD.

## 1. Introduction

Adult Onset Still's Disease (AOSD) is a rare systemic inflammatory disease of unknown etiology.It is characterized by a typical clinical triad: high daily spiking fever, evanescent rash and arthritis or arthralgias, not necessarily present at the same time. A wide spectrum of other symptoms may occur: sore throat, lymphoadenopathy, hepatosplenomegaly, serositis, and myalgias; also pulmonary, cardiovascular, and kidney manifestations may be present occasionally representing severe life-threatening complications [1]. AOSD has been described for the first time by Bywaters in 1971 [2] who included in this entity all the patients that did not meet criteria of Rheumatoid Arthritis (RA) but showing the typical manifestations of the systemic form of juvenile rheumatoid arthritis. Usually, the disease onset is between 16–35 years [3], women are slightly more affected (M/F = 40/60) [4], while men have an earlier onset [5]. AOSD pathogenesis is still controversial: on a background of genetic predisposition, different infectious factors seem to act as disease triggers. Moreover, immunological factors are involved: it has been recently suggested that an imbalance in cytokine production by T helper 1 (Th1) versus T helper 2 (Th2) cells is a key mechanism. In particular, a major production of Th1 cytokines as interleukin (IL)-2, interferon (IFN)-*γ* and TNF-*α* compared with Th2 cytokines (IL-4, IL-5, IL-6 e IL-10) has been demonstrated [6]. Among all cytokines, IL-18 seems to play a pivotal role being overexpressed during the course of disease [7]. However, whether its serum level represents a marker of disease activity is still controversial [8]. Thus, the aim of this study was to determine IL-18 serum levels in a cohort of Italian patients with active and non-active AOSD, to compare these levels with those obtained from patients affected with other inflammatory diseases and to correlate them with other known markers of disease activity. 

## 2. Methods

Consecutive patients with AOSD (diagnosed according to Yamaguchy criteria [9]) followed during the last five years in the Rheumatology Unit of Sapienza, University of Rome (Italy), were enrolled. The patients underwent clinical evaluation and laboratory analysis. Sera were collected for IL-18 analysis which was performed by means of ELISA test (Immuno Pharmacology Research, Italy). 

Disease activity at the moment of drawing was estimated using the criteria proposed by Pouchot in 1991 [10]. The total score ranges from 0 to 12 and is calculated through the addition of points assigned to each symptom (fever, evanescent rash, pleuritis, pneumonia, pericarditis, hepatomegaly or abnormal liver function tests, splenomegaly, lymphadenopathy, WBC > 15000/mm^3^, sore throat, myalgias, and abdominal pain). Active patients were considered those who presented fever at the moment of drawing with a Pouchot's score > 2. For each patient serum ferritin and C-reactive protein (CRP) serum levels were also determined. Twenty-one patients with RA, 21 patients with Sjogren's Syndrome (SS), 20 patients with Systemic Lupus Erythematosus (SLE), and 21 healthy subjects (normal human sera, NHS) were included in this study as control groups. All subjects, patients and controls, provided their informed consent. 

For the statistical analysis Mann-Whitney *U* test and Spearman's rank correlation test were used. Two-tailed *P* values less than 0.05 were considered significant. Area under the receiver operating characteristic curve (ROC-AUC) analysis was used to evaluate the diagnostic utility of the IL-18 serum level.

## 3. Results

Twenty-six patients with AOSD were enrolled (15 males/11 females; mean age 40.6 years, range 23–69 years; mean age at disease's onset 32.9 years, range 12–55 years). 

Mean disease activity score according to Pouchot's criteria was 3.8; 16/26 (61%) patients were considered active presenting fever and a Pouchot's score > 2. Serum IL-18 mean value in the whole cohort was 461.33 pg/mL (range 20.74–6015.00 pg/mL). 

**Figure 1:**
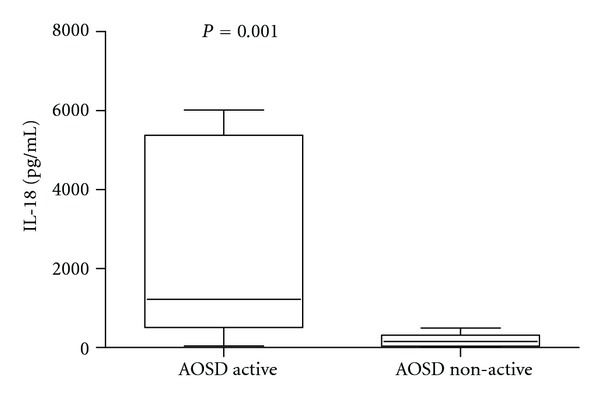
Box-and-whisker plot of IL-18 serum levels in patients with active AOSD (*n* = 16) and non-active AOSD (*n* = 10).

**Figure 2:**
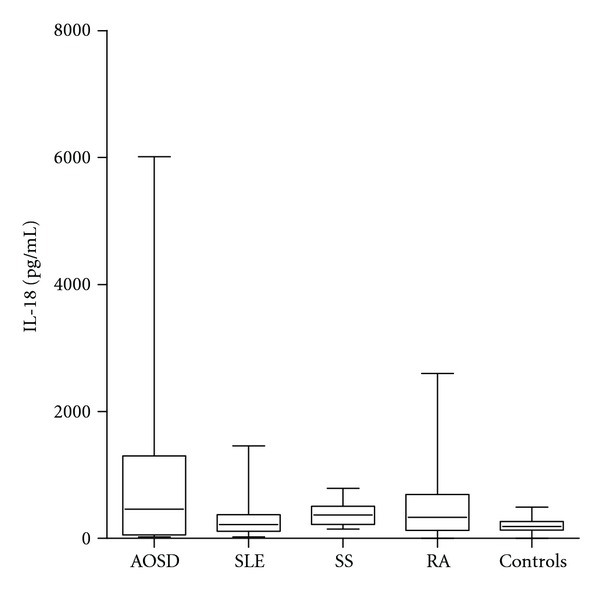
Box-and-whisker plot of IL-18 serum levels in patients with AOSD (*n* = 26), RA (*n* = 21) SS (*n* = 21), SLE (*n* = 20), and NHS (*n* = 21). Median, quartiles, range, and possibly extreme values are shown.

**Figure 3:**
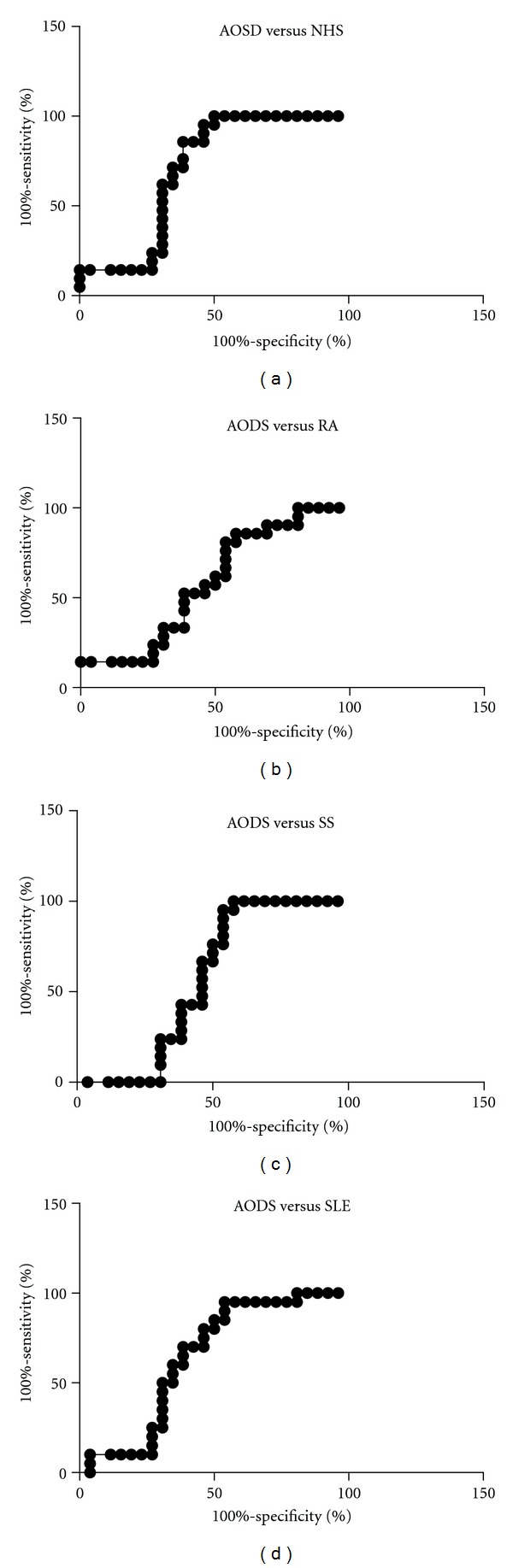
Area under the receiver operating characteristic curves for detection of AOSD by reference to the level of serum IL-18. (a) AOSD versus NHS; (b) AOSD versus RA; (c) AOSD versus SS; (d)AOSD versus SLE.

IL-18 was significantly higher in patients with active AOSD than non-active AOSD (*P* = 0.001) (Figure 1). Moreover IL-18 was significantly higher in patients with active AOSD compared with the other control groups (RA *P* = 0.0070, SS *P* = 0.0029, SLE *P* = 0.0032, NHS *P* = 0.0004) (Figure 2, Table 1). Thirteen on 26 (50%) patients with AOSD and 12/16 (75%) with active AOSD showed values of serum IL-18 greater than the highest IL-18 value detected from NHS group. ROC-AUC analysis of the serum concentration of IL-18 indicated that it was significantly diagnostic of AOSD. The ROC-AUC analysis for the serum level of the IL-18 between patients with AOSD and NHS was 0.701 (Figure 3(a)). At a cutoff point of 312.5 pg/mL, corresponding to the greatest sum of specificity and sensitivity, the specificity was 61.54% and the sensitivity was 86.71% for detection of AOSD (likelihood 2.23). The ROC-AUC analysis for the serum level of the IL-18 between patients with AOSD and the other control groups (RA, SS, SLE) was, respectively, 0.586, 0.565, 0.640 (Figures 3(b), 3(c), and 3(d)). For RA at a cutoff point of 737 pg/mL, specificity was 46.15%, sensitivity 80.95% (likelihood 1.50). For SS at a cutoff point of 766 pg/mL specificity was 46.15%, sensitivity 95.24% (likelihood 1.77). For SLE at a cutoff point of 336 pg/mL specificity was 61.54%, sensitivity 70% (likelihood 1.82).

**Figure 4:**
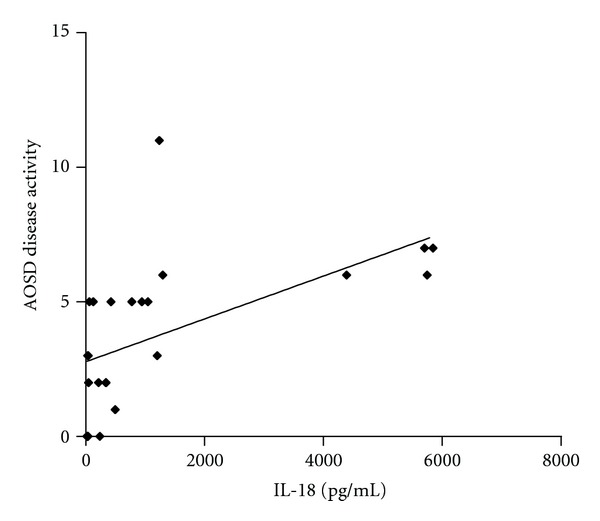
Correlation between disease activity and IL-18 serum levels.

**Figure 5:**
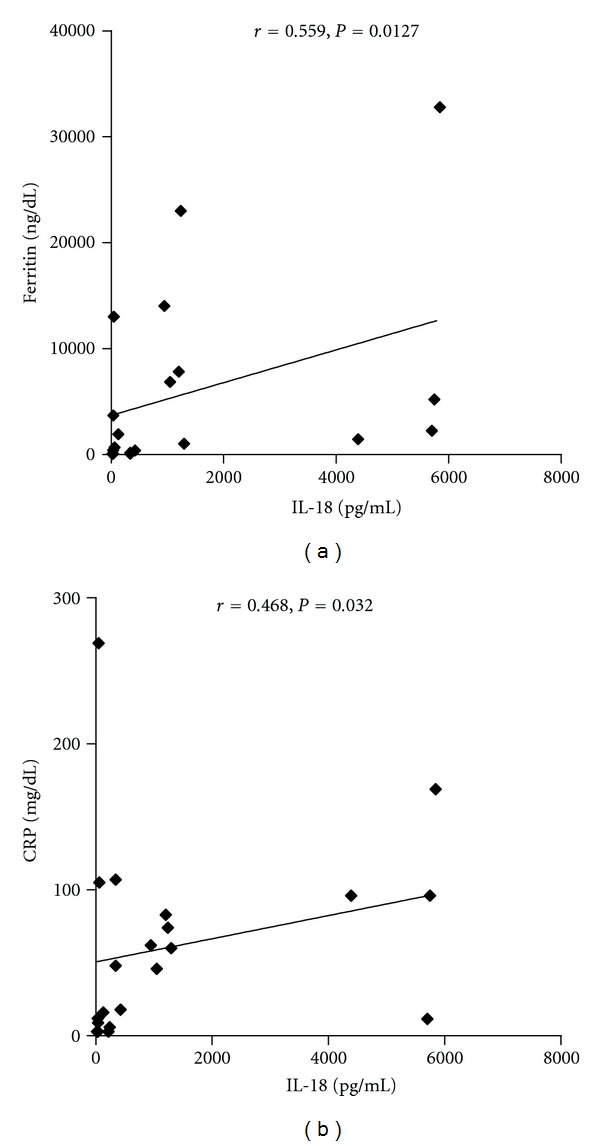
(a) Correlation between CRP serum levels and IL-18 serum levels. (b) Correlation between serum ferritin levels and IL-18 serum levels.

A significant correlation between disease activity and IL-18 serum levels was observed (*P* < 0.0001, by Spearman's rank correlation test) (Figure 4), as well as with serum ferritin level and CRP (*P* = 0.0127 and *P* = 0.0032, resp., by Spearman's rank correlation test) (Figures 5(a), and 5(b)).

## 4. Discussion

We confirm the key role of IL-18 as a marker of disease activity in AOSD. High serum levels of IL-18 are detected in AOSD patients with an active disease and its concentration correlates with disease activity and laboratory features of inflammation. IL-18 is a proinflammatory cytokine considered a member of the IL-1 superfamily with pleiotropic and immunoregulatory effects. It plays a pivotal role in different inflammatory diseases such as RA, Crohn's disease (CD), SS, and psoriatic arthritis (PsA) as well as AOSD [7]. It has been described for the first time in 1989 as an inducer of the production of IFN-*γ*. In synergy with IL-12 and IL-15, it induces Th1 maturation and production of other cytokines as IL-1*β*, IL-8, M-CSF, TNF-*α*, and IFN-*γ*. Despite its prevalent role on Th1 lineage, it seems to induce and promote Th2 mediated-effects depending on the different milieu in which it plays [11]. Beside this role in the regulation of adaptive immune responses, it is involved in inducing innate immunity. Indeed, it is produced by dendritic cells (DCs) providing natural killer (NK) cells activation and driving IFN-*γ* production by these cells. In turn, NK cells induce DCs maturation with a further activation of the adaptive immune response. Thus, IL-18 provides a critical link between adaptive and innate immune responses and, for this reason, it can be considered as a rather unique cytokine [7]. Furthermore, IL-18 can drive angiogenesis [12] and the production of chemokines, it can upregulate the expression of costimulatory molecules as CD40 and CD40L, and, finally, it can provoke tissue damage through the activation of cell-mediated cytotoxicity and the stimulation of the release of metalloproteases [7]. It is also responsible of an increased expression of the intercellular adhesion molecule-1 (ICAM-1) by endothelial cells, representing a possible predictor of disseminated intravascular coagulation [13]. Moreover, a recent study has proposed a proapoptotic role for IL-18 that has been associated with the induction of an increased expression of FasL and p53 on autoreactive lymphocytes in AOSD [14]. This evidence is supported by the demonstration of increased apoptosis of peripheral blood lymphocytes in AOSD that would be also correlated with disease activity [14]. This mechanism is deputed to balance the excess of auto-reactive cells present in peripheral blood in AOSD, which is responsible of the chronic inflammatory process. IL-18 is produced not only by monocyte/macrophages and DCs, but also by nonimmune cells, such as intestinal epithelial cells, keratinocytes, chondrocytes, osteoblasts, and synovial fibroblasts [7]. For this reason, high values of IL-18 are detectable not only in serum but also in the synovial fluid. It has been shown from studies on synovial biopsies that in AOSD there is an important inflammatory process characterized by the presence of synoviocytes proliferation with lymphocytes and plasmacells infiltrate. Moreover, at this level, high expression of mRNA codifying for IL-18 can been detected [15]. 

In previous studies, we have shown that IL-18 is over-expressed also in other sites, such as lymph nodes, and liver [16, 17]. Indeed, an hepatic inflammatory process can occur in approximately 50–75% of the patients, with hepatomegaly and liver enzymes elevation. It is not surprising that IL-18 can be produced by Kupffer cells and can provoke liver damage through the activation of NK cells and CD8 cells [18].

As above mentioned, IL-18 has a pivotal role in several other inflammatory conditions such as RA, CD, SS, and PsA. In RA, high expression of IL-18 by synoviocytes may upregulate the production of pathogenic cytokines responsible for the local inflammatory process. In SS, IL-18 is produced by infiltrating immune cells surrounding the ductal structures of the salivary glands, and high levels of the cytokine have been shown in mucosal biopsies from CD patients, and in psoriatic lesional skin [7]. Nevertheless, serum IL-18 reaches the highest levels in AOSD, even from 100 to 1000 folds than in other diseases. 

ROC-AUC analysis revealed that AOSD patients could be discriminated from RA, SLE, and SS patients and from healthy subjects by the serum level of IL-18 determined by ELISA. As expected, the largest ROC area was obtained when comparing AOSD and healthy controls. However, at higher cutoff points, IL-18 seems to be able to discriminate also with diseases which have increased levels of this cytokine as RA and SS. These results may suggest that the level of the IL-18 in serum has potential clinical application as a biomarker for the diagnosis or differential diagnosis of AOSD. 

It has been showed that IL-18 serum levels correlate with other serological markers of disease activity, such as ferritin, erythrocytes sedimentation rate, and CRP, and with clinical manifestations as the occurrence of fever and arthralgias [17]. On the contrary, Choi coll. [8] did not demonstrate any significant differences between patients with active and inactive AOSD, thus the role of IL-18 as a marker of disease activity is still controversial. In this study a significant correlation between IL-18 and some laboratory markers of disease activity (ferritin and CRP serum levels) was found. Indeed, it is well known that serum ferritin levels are typically increased during disease flares and this finding is useful for diagnosis and monitoring of disease [1]. Kawashima et al. in 2001 determined IL-18 serum levels in 16 patients with AOSD, 34 with RA, 33 with SLE, 19 with systemic sclerosis (SSc), 21 with polymyositis/dermatomyositis, 28 with SS and in 53 healthy controls demonstrating, as we did in the present report, that IL-18 serum concentrations are higher in AOSD than in all the other conditions [19]. According to Kawaguchi and coll., IL-18 may be also considered a marker able to predict the response to therapy [20]. These authors have observed how patients considered responders to corticosteroids therapy present lower cytokine levels in the peripheral blood than nonresponders. This finding is in agreement with our observation that IL-18 levels correlate with disease activity, suggesting that the level may gradually decrease with therapy.

Nowadays, AOSD therapy is still mostly empirical and Disease Modifying Antirheumatic Drugs are considered the first-line therapy. In patients refractory to traditional therapeutic approach, biologic agents have been used with successful results [21, 22]. Anti-TNF therapy and anti-IL1 and anti-IL-6 agents have been used in an off-label fashion with a generally good outcome. Nonetheless, no controlled randomized trial is available yet. This paper, in agreement with previous reports, provides further evidence to support the targeting of IL-18 as a possible therapeutic strategy in AOSD. 

## Figures and Tables

**Table 1:** IL-18 serum levels in patients with Adult Onset Still's Disease (AOSD), Rheumatoid Arthritis (RA), Sjogren's Syndrome (SS), Systemic Lupus Erythematosus (SLE), and in normal human sera (NHS).

	Active AOSD (*n *= 16)	RA (*n *= 21)	SS (*n *= 21)	SLE (*n *= 20)	NHS (*n *= 21)
IL-18 (pg/mL)	1220.47	331	368	218.5	189
Range (pg/mL)	43.80–6010	0–2600	147–788	22–1459	0–492

## References

[1] Priori R, Colafrancesco S, Gattamelata A, di Franco M, di Tondo U, Valesini G (2010). Adult-onset still disease: a rare disorder with a potentially fatal outcome. *Autoimmunity Highlights*.

[2] Bywaters EG (1971). Still’s disease in the adult. *Annals of the Rheumatic Diseases*.

[3] Ohta A, Yamaguchi M, Kaneoka H, Nagayoshi T, Hiida M (1988). Adult Still’s disease: review of 228 cases from the literature. *Journal of Rheumatology*.

[4] Wakai K, Ohta A, Tamakoshi A (1997). Estimated prevalence and incidence of adult Still’s disease: findings by a nationwide epidemiological survey in Japan. *Journal of Epidemiology*.

[5] Ohta A, Yamaguchi M, Tsunematsu T (1990). Adult Still’s disease: a multicenter survey of Japanese patients. *Journal of Rheumatology*.

[6] Chen DY, Lan JL, Lin FJ, Hsieh TY, Wen MC (2004). Predominance of Th1 cytokine in peripheral blood and pathological tissues of patients with active untreated adult onset Still’s disease. *Annals of the Rheumatic Diseases*.

[7] Bombardieri M, McInnes IB, Pitzalis C (2007). Interleukin-18 as a potential therapeutic target in chronic autoimmune/inflammatory conditions. *Expert Opinion on Biological Therapy*.

[8] Choi JH, Suh CH, Lee YM (2003). Serum cytokine profiles in patients with adult onset Still's disease. *Journal of Rheumatology*.

[9] Yamaguchi M, Ohta A, Tsunematsu T (1992). Preliminary criteria for classification of adult Still’s disease. *Journal of Rheumatology*.

[10] Pouchot J, Sampalis JS, Beaudet F (1991). Adult Still’s disease: manifestations, disease course, and outcome in 62 patients. *Medicine*.

[11] Reddy P (2004). Interleukin-18: recent advances. *Current Opinion in Hematology*.

[12] Dai SM, Nishioka K, Yudoh K (2004). Interleukin (IL) 18 stimulates osteoclast formation through synovial T cells in rheumatoid arthritis: comparison with IL1*β* and tumour necrosis factor *α*. *Annals of the Rheumatic Diseases*.

[13] Chen DY, Lan JL, Lin FJ, Hsieh TY (2005). Association of intercellular adhesion molecule-1 with clinical manifestations and interleukin-18 in patients with active, untreated adult-onset Still’s disease. *Arthritis Care and Research*.

[14] Chen DY, Hsieh TY, Hsieh CW, Lin FJ, Lan JL (2007). Increased apoptosis of peripheral blood lymphocytes and its association with interleukin-18 in patients with active untreated adult-onset Still’s disease. *Arthritis Care and Research*.

[15] Chen DY, Lan JL, Lin FJ, Hsieh TY (2004). Proinflammatory cytokine profiles in sera and pathological tissues of patients with active untreated adult onset still’s disease. *Journal of Rheumatology*.

[16] Conigliaro P, Priori R, Bombardieri M (2009). Lymph node IL-18 expression in adult-onset Still’s disease. *Annals of the Rheumatic Diseases*.

[17] Priori R, Barone F, Alessandri C (2011). Markedly increased IL-18 liver expression in adult-onset Still’s disease-related hepatitis. *Rheumatology*.

[18] Tsutsui H, Matsui K, Okamura H, Nakanishi K (2000). Pathophysiological roles of interleukin-18 in inflammatory liver diseases. *Immunological Reviews*.

[19] Kawashima M, Yamamura M, Taniai M (2001). Levels of interleukin-18 and its binding inhibitors in the blood circulation of patients with adult-onset still’s disease. *Arthritis & Rheumatism*.

[20] Kawaguchi Y, Terajima H, Harigai M, Hara M, Kamatani N (2001). Interleukin-18 as a novel diagnostic marker and indicator of disease severity in adult-onset Still’s disease. *Arthritis & Rheumatism*.

[21] Priori R, Ceccarelli F, Barone F, Iagnocco A, Valesini G (2008). Clinical, biological and sonographic response to IL-1 blockade in adult-onset still’s disease. *Clinical and Experimental Rheumatology*.

[22] Franchini S, Dagna L, Salvo F, Aiello P, Baldissera E, Sabbadini MG (2010). Efficacy of traditional and biologic agents in different clinical phenotypes of adult-onset Still’s disease. *Arthritis and Rheumatism*.

